# Genomes or exomes: evaluation of cost, time and coverage

**DOI:** 10.1186/gb-2011-12-s1-p43

**Published:** 2011-09-19

**Authors:** Sumit Middha, Jeanne L Theis, Adele H Goodloe, Timothy M Olson, Jean-Pierre A Kocher

**Affiliations:** 1 Division of Biomedical Statistics and Informatics, Mayo Clinic, Rochester, MN, USA; 2 Division of Cardiovascular Diseases, Mayo Clinic, Rochester, MN, USA

## 

Next-generation sequencing technology platforms are driving the development of a variety of approaches to study genomic variation associated with disease. One of these approaches, exome sequencing, specifically targets the coding regions of the genome, which are captured and sequenced. Compared with whole genome sequencing, exome sequencing offers the advantages of being cost- and time-effective while providing deeper coverage of coding variants, which are more likely to affect function.

However, the protocol is known to be only partially reliable and might miss some of the coding regions. To assess how much coding region could be missed or of target, we compared whole genome and exome sequencing data derived from one sample that was processed by the Illumina GA-IIx platform.

Our in-house-developed workflow named TREAT (Targeted RE-sequencing and Annotation Tool) was used to align and annotate the data. We provide a summary of the comparison between the two datasets, including the total number of reads produced, the time needed for sequencing and analysis, the coverage of coding regions and the agreement between called variants.

